# Supplementary material for: Optimization and Spectrum–Effect Analysis of Ultrasonically Extracted Antioxidant Flavonoids from Persicae Ramulus
Source: Molecules. 2024 Aug 15;29(16):3860. doi: 10.3390/molecules29163860 (PMC11356933; doi:10.3390/molecules29163860)
Supplement: Supplementary file 1 [file molecules-29-03860-s001.zip › molecules-3091434-supplementary.pdf]

contents

Table S1. Validation of method for flavonoid determination (n=3) 2
Table S2. Results of single - factor tests (n=3) 2
Table S3. Response surface experiment designs and results of flavonoids from persicae ramulus (n=3) 2
Table S4. Analysis of variance of regression model 3
Table S5. Yield of flavonoids extract in different batches of persicae ramulus (n=3) 3
Table S6. Clearance of DPPH and ABTS free radicals in twenty-eight batches of persicae ramulus ($\overline{X}$±SD) (n=3) 3
Table S7. Gradient elution conditions of mobile phase 4
Table S8. Method precision, stability, and repeatability data 5
Table S9. RRT of common peaks of twenty-eight batches of samples. 6
Table S10. RPA of common peaks of twenty-eight batches of samples 9
Table S11. Eigenvalues and contribution rates of twenty-eight samples 12
Table S12. Loading matrix analysis results of 35 common peaks' principal components in twenty-eight samples. 13
Table S13. Score coefficient matrix of the chemical constituents. 14
Table S14. Principal component score result 16
Table S15. VIP values of 35 chromatographic peaks with antioxidant activity 18
Table S16. Regression coefficients of 35 common peaks with antioxidant activity. 18
Table S17. Twenty-eight batches of persicae ramulus from different areas. 19
Table S18. Factors and levels of process optimization of RSM 19
Figure S1. System suitability investigation results (UPLC conditions of persicae ramulus; a: column; b: wavelength; c: analysis time; d: mobile phase; e: flow rate; f: column temperature) 20
Figure S2. Methodology validation of UPLC (a: precision; b: reproducibility; c: stability; d: blank) 21
Figure S3. Standard curve 22

Table S1. Validation of method for flavonoid determination (n=3)

	Mean absorbance	Yield of flavonoids (mg/g)	RSD%
Precision	0.62	3.04±0.01	0.13
Stability	0.61	2.97±0.06	2.18
Repeatability	0.62	3.02±0.01	0.57
Recovery	1.10	96.86±2.83	2.93

Note: n=3 was triplicate repetitions of parallel trials.

Table S2. Results of single-factor tests (n=3)

Factors		Yield of flavonoids (mg/g)
Time	30	2.48±0.04
	40	2.53±0.02
	50	3.01±0.04
	60	2.48±0.07
	70	2.45±0.03
Solid-to-liquid ratio	15	2.52±0.03
	20	2.72±0.02
	25	3.10±0.02
	30	3.00±0.03
	35	2.73±0.01
Volume fraction of ethanol	30	2.67±0.02
	40	2.94±0.04
	50	2.52±0.03
	60	2.43±0.02
	70	1.94±0.02

Note: n=3 was triplicate repetitions of parallel trials.

Table S3. Response surface experiment designs and results of flavonoids from persicae ramulus (n=3)

Trial number	levels			Y
	A (min)	B (g/mL)	C (%)	Yield of flavonoids (mg/g)
1	40	20	50	2.18±0.01
2	60	20	50	2.31±0.13
3	40	30	50	2.72±0.03
4	60	30	50	2.53±0.06
5	40	25	40	2.80±0.03
6	60	25	40	2.83±0.11
7	40	25	60	2.94±0.10
8	60	25	60	3.02±0.08
9	50	20	40	2.08±0.17
10	50	30	40	2.58±0.19
11	50	20	60	2.17±0.06
12	50	30	60	3.01±0.05
13	50	25	50	3.25±0.09
14	50	25	50	3.27±0.03
15	50	25	50	3.27±0.06
16	50	25	50	3.34±0.09

17	50	25	50	3.39±0.06

Note: A was extraction time; B was solid-liquid ratio; c was volume fraction of ethanol, n=3 was triplicate repetitions of parallel trials.

Table S4. Analysis of variance of regression model

Soure	Square deviation sum	Degree of freedom	F value	P value	Significance
Model	3	9	35.74	< 0.01	**
A	1.91×10^{-4}	1	0.02	0.89	
B	0.55	1	58.96	0.01×10^{-2}	**
C	0.1	1	9.81	0.02	*
AB	0.03	1	2.79	0.14	
AC	3.81×10^{-4}	1	0.04	0.85	
BC	0.03	1	3.13	0.12	
A^2	0.19	1	20.84	0.26×10^{-2}	**
B^2	1.8	1	192.5	< 0.01	**
C^2	0.16	1	16.68	0.47×10^{-2}	**
Residuals	0.07	7			
Lack of Fit	0.05	3	5.2	0.73×10^{-1}	no
Pure Error	0.01	4			
Cor Total	3.07	16			

Note: * $p < 0.05$ in the table was significant on statistics, ** $p < 0.01$ was extremely significant on statistics.

Table S5. Yield of flavonoids extract in different batches of persicae ramulus (n=3)

Batch	Yield of flavonoids (mg/g)	Batch	Yield of flavonoids (mg/g)
S1	3.76±0.03	S15	1.34±0.01
S2	1.96±0.04	S16	0.80±0.03
S3	2.83±0.01	S17	1.33±0.06
S4	3.08±0.02	S18	1.88±0.01
S5	3.62±0.05	S19	1.77±0.04
S6	2.90±0.02	S20	2.05±0.16
S7	3.76±0.11	S21	0.74±0.01
S8	3.24±0.01	S22	1.64±0.03
S9	2.34±0.03	S23	2.15±0.04
S10	2.09±0.02	S24	1.38±0.02
S11	2.63±0.04	S25	2.27±0.04
S12	3.31±0.35	S26	1.56±0.06
S13	2.16±0.03	S27	0.93±0.12
S14	2.37±0.03	S28	1.52±0.11

Note: n=3 was the number of repetitions of parallel trials.

Table S6. Clearance of DPPH and ABTS free radicals in twenty-eight batches of persicae ramulus ($\overline{X}$±SD) (n=3)

Batch	DPPH (%)	ABTS (%)	Batch	DPPH (%)	ABTS (%)
S1	85.33±0.8	66.40±1.27	S15	53.92±0.71	38.86±0.74
S2	58.65±0.32	40.33±1.32	S16	40.19±0.37	24.73±0.49
S3	86.56±1.32	60.17±0.28	S17	53.48±0.66	41.86±0.62
S4	87.89±0.63	70.84±0.32	S18	79.23±0.66	70.65±0.24
S5	87.64±0.69	68.90±1.39	S19	67.94±0.87	49.00±0.33
S6	73.60±1.73	46.35±1.10	S20	80.67±1.20	70.46±0.22
S7	87.51±0.62	62.70±0.50	S21	66.80±1.15	87.56±0.40
S8	58.24±0.36	50.68±1.53	S22	78.71±0.24	59.86±0.29
S9	79.49±1.04	61.65±0.62	S23	62.55±1.12	55.67±0.91
S10	70.33±0.47	56.37±0.62	S24	80.06±0.17	59.41±0.63
S11	83.31±0.89	66.71±0.42	S25	82.74±0.37	73.67±0.55
S12	79.49±0.31	70.78±0.78	S26	69.21±0.73	57.65±0.76
S13	81.10±0.63	52.03±1.21	S27	75.21±0.82	59.54±0.79
S14	82.05±0.22	73.94±1.03	S28	71.20±0.64	71.86±0.24

Note: n=3 was the number of repetitions of parallel trials.

Table S7. Gradient elution conditions of mobile phase

Time（min）	A（%）	B（%）
0	90	10
5	87	13
15	78	22
20	70	30
25	60	40
30	1	99

Table S8. Method precision, stability, and repeatability data

Common peaks	Precision				Stability				Repeatability			
	RRT	RSD (%)	RPA	RSD (%)	RRT	RSD (%)	RPA	RSD (%)	RRT	RSD (%)	RPA	RSD (%)
1	0.05	0.35	0.30	0.69	0.05	0.27	0.30	0.69	0.05	0.74	0.30	1.23
2	0.07	0.38	0.05	0.47	0.07	0.27	0.05	1.69	0.07	0.76	0.05	1.53
3	0.10	0.48	0.03	1.40	0.10	0.34	0.03	1.92	0.10	1.89	0.03	1.71
4	0.12	2.51	0.01	1.22	0.12	0.30	0.01	1.79	0.12	1.80	0.01	1.75
5	0.16	2.53	0.11	1.24	0.15	0.46	0.11	1.23	0.15	2.05	0.11	1.28
6	0.21	2.72	3.48	0.47	0.22	0.65	3.45	0.28	0.22	1.78	3.43	0.39
7	0.28	1.86	0.25	1.45	0.28	0.64	0.24	1.55	0.28	2.35	0.24	0.92
8	0.29	1.57	0.07	1.74	0.30	0.72	0.07	0.94	0.30	2.62	0.07	1.76
9	0.35	1.63	0.70	0.69	0.37	0.58	0.70	0.44	0.37	1.88	0.67	1.23
10	0.50	1.38	0.09	1.37	0.49	0.51	0.09	0.71	0.48	1.59	0.06	1.95
11	0.57	1.00	0.19	0.75	0.58	0.42	0.19	0.29	0.58	1.55	0.19	0.37
12	0.63	0.91	0.02	1.22	0.63	0.38	0.02	0.44	0.63	1.54	0.02	1.23
13	0.65	0.66	0.03	1.92	0.64	0.31	0.03	1.11	0.64	1.44	0.04	1.32
14	0.67	0.52	0.09	0.89	0.68	0.27	0.09	0.91	0.68	1.38	0.10	0.77
15	0.78	0.57	0.07	0.13	0.78	1.79	0.07	0.57	0.79	0.12	0.07	1.10
16	0.85	0.14	0.39	0.33	0.85	0.06	0.41	0.41	0.85	0.42	0.40	1.68
17	1.00	0.00	0.41	0.45	1.00	0.00	1.00	0.00	1.00	0.00	0.41	0.37
18	1.10	0.10	1.00	0.00	1.10	0.05	1.43	0.05	1.09	0.04	1.00	0.00
19	1.12	0.46	1.40	1.23	1.12	0.10	0.03	0.80	1.12	0.39	1.45	1.30
20	1.15	0.44	0.02	1.88	1.16	0.07	0.08	0.68	1.16	0.31	0.03	1.24
21	1.18	0.16	0.08	1.38	1.18	0.05	0.04	0.23	1.18	0.16	0.08	0.32
22	1.22	0.31	0.04	0.46	1.21	0.07	0.16	0.39	1.21	0.10	0.04	0.40

23	1.31	0.61	0.18	1.93	1.30	0.08	0.35	0.20	1.29	0.94	0.16	0.25
24	1.33	0.68	0.35	0.18	1.34	0.08	0.01	1.93	1.34	0.11	0.35	0.11
25	1.38	0.89	0.01	1.96	1.37	0.08	0.34	0.20	1.36	0.69	0.01	1.82
26	1.49	0.35	0.34	0.57	1.49	0.12	2.48	0.07	1.48	0.64	0.34	1.67
27	1.76	0.34	2.48	0.12	1.75	0.19	0.02	1.03	1.75	0.40	2.49	0.58
28	1.80	0.35	0.02	1.70	1.80	0.19	0.23	0.30	1.79	0.89	0.02	1.51
29	1.92	0.09	0.23	0.33	1.92	0.20	0.00	1.96	1.92	0.75	0.23	0.93
30	1.94	0.10	0.00	1.96	1.95	0.21	0.03	0.26	1.94	0.74	0.00	1.46
31	1.97	0.11	0.03	0.62	1.97	0.21	0.00	1.98	1.96	0.72	0.03	0.45
32	2.02	0.13	0.05	0.27	2.02	0.22	0.05	0.37	2.01	0.64	0.05	1.33
33	2.19	0.41	0.20	0.38	2.18	0.24	0.20	0.26	2.18	0.28	0.20	0.35
34	2.24	0.44	0.07	0.17	2.23	0.26	0.07	0.05	2.23	0.71	0.08	1.84
35	2.26	0.14	0.01	1.19	2.28	0.75	0.00	1.84	2.27	0.65	0.04	1.34

Note: RRT was relative retention times

Table S9. RRT of common peaks of twenty-eight batches of samples.

Peak	S1	S2	S3	S4	S5	S6	S7	S8	S9	S10	S11	S12	S13	S14
1	0.06	0.06	0.06	0.06	0.06	0.06	0.06	0.06	0.06	0.06	0.06	0.06	0.06	0.06
2	0.09	0.08	0.08	0.08	0.08	0.08	0.08	0.08	0.08	0.08	0.08	0.08	0.08	0.08
3	0.10	0.11	0.11	0.11	0.11	0.11	0.11	0.11	0.11	0.11	0.11	0.11	0.11	0.11
4	0.12	0.13	0.13	0.13	0.13	0.13	0.13	0.13	0.13	0.14	0.13	0.13	0.13	0.13
5	0.17	0.18	0.17	0.18	0.18	0.17	0.18	0.18	0.18	0.18	0.17	0.18	0.18	0.18
6	0.21	0.22	0.21	0.21	0.21	0.21	0.21	0.21	0.22	0.22	0.21	0.21	0.21	0.21
7	0.24	0.26	0.25	0.25	0.25	0.25	0.25	0.26	0.26	0.26	0.25	0.25	0.25	0.25
8	0.28	0.30	0.29	0.29	0.29	0.29	0.29	0.29	0.29	0.30	0.29	0.29	0.29	0.29
9	0.36	0.38	0.38	0.38	0.38	0.38	0.38	0.38	0.38	0.38	0.38	0.38	0.38	0.38
10	0.47	0.50	0.49	0.49	0.49	0.49	0.49	0.49	0.49	0.50	0.49	0.49	0.49	0.49

11	0.57	0.57	0.56	0.56	0.56	0.56	0.56	0.56	0.56	0.57	0.56	0.56	0.56	0.56
12	0.61	0.60	0.59	0.59	0.60	0.60	0.60	0.60	0.60	0.60	0.59	0.59	0.59	0.59
13	0.66	0.64	0.64	0.63	0.64	0.64	0.64	0.64	0.63	0.64	0.63	0.63	0.64	0.64
14	0.69	0.68	0.68	0.68	0.68	0.68	0.68	0.68	0.68	0.68	0.68	0.68	0.68	0.68
15	0.76	0.75	0.75	0.75	0.75	0.75	0.75	0.75	0.75	0.76	0.75	0.75	0.75	0.75
16	0.84	0.85	0.85	0.85	0.86	0.86	0.86	0.86	0.86	0.85	0.81	0.81	0.81	0.86
17	1.00	1.00	1.00	1.00	1.00	1.00	1.00	1.00	1.00	1.00	1.00	1.00	1.00	1.00
18	1.07	1.06	1.06	1.06	1.06	1.06	1.06	1.06	1.06	1.06	1.06	1.06	1.06	1.06
19	1.11	1.10	1.10	1.10	1.10	1.10	1.10	1.10	1.10	1.10	1.10	1.10	1.10	1.10
20	1.15	1.15	1.15	1.15	1.14	1.15	1.15	1.15	1.15	1.15	1.15	1.15	1.15	1.15
21	1.21	1.20	1.20	1.20	1.20	1.20	1.20	1.20	1.20	1.20	1.20	1.20	1.20	1.20
22	1.25	1.24	1.24	1.24	1.24	1.24	1.24	1.24	1.24	1.24	1.24	1.24	1.24	1.24
23	1.31	1.30	1.30	1.30	1.30	1.30	1.30	1.30	1.30	1.30	1.30	1.30	1.30	1.30
24	1.35	1.34	1.34	1.34	1.34	1.34	1.34	1.34	1.34	1.34	1.34	1.34	1.34	1.34
25	1.38	1.37	1.37	1.37	1.36	1.37	1.37	1.36	1.37	1.36	1.37	1.37	1.36	1.36
26	1.51	1.50	1.50	1.49	1.49	1.49	1.50	1.50	1.50	1.49	1.50	1.50	1.49	1.50
27	1.72	1.71	1.70	1.70	1.70	1.70	1.66	1.70	1.70	1.70	1.70	1.70	1.70	1.70
28	1.77	1.76	1.76	1.76	1.75	1.76	1.70	1.76	1.76	1.76	1.76	1.76	1.76	1.76
29	1.81	1.80	1.79	1.80	1.79	1.80	1.76	1.80	1.80	1.80	1.80	1.80	1.80	1.80
30	1.85	1.84	1.84	1.84	1.83	1.84	1.80	1.84	1.84	1.84	1.84	1.84	1.84	1.84
31	1.88	1.87	1.86	1.87	1.86	1.86	1.84	1.86	1.86	1.86	1.87	1.87	1.87	1.87
32	1.93	1.92	1.92	1.92	1.91	1.92	1.92	1.92	1.91	1.91	1.92	1.92	1.92	1.92
33	2.01	2.00	2.00	2.00	1.99	2.00	2.00	2.00	2.00	1.99	2.00	2.00	2.00	2.00
34	2.16	2.15	2.15	2.15	2.14	2.15	2.15	2.15	2.15	2.15	2.16	2.16	2.15	2.16
35	2.20	2.19	2.19	2.19	2.19	2.19	2.20	2.20	2.19	2.19	2.20	2.20	2.20	2.20

RRT of common peaks of twenty-eight batches of samples (continuation table)

Peak	S15	S16	S17	S18	S19	S20	S21	S22	S23	S24	S25	S26	S27	S28
1	0.06	0.06	0.06	0.06	0.06	0.06	0.06	0.06	0.06	0.06	0.06	0.06	0.06	0.06
2	0.08	0.08	0.08	0.08	0.08	0.08	0.08	0.08	0.08	0.08	0.08	0.08	0.08	0.08
3	0.11	0.11	0.11	0.11	0.11	0.11	0.11	0.11	0.11	0.11	0.11	0.11	0.11	0.11
4	0.13	0.13	0.14	0.13	0.13	0.14	0.13	0.14	0.13	0.14	0.13	0.13	0.13	0.14
5	0.18	0.18	0.18	0.17	0.18	0.18	0.18	0.18	0.18	0.17	0.18	0.17	0.17	0.17
6	0.21	0.21	0.22	0.21	0.21	0.22	0.22	0.21	0.21	0.21	0.21	0.21	0.21	0.20
7	0.25	0.25	0.26	0.25	0.26	0.25	0.26	0.25	0.26	0.25	0.26	0.25	0.25	0.23
8	0.29	0.28	0.29	0.29	0.29	0.29	0.29	0.29	0.29	0.29	0.29	0.29	0.29	0.28
9	0.38	0.38	0.38	0.38	0.38	0.38	0.38	0.38	0.38	0.38	0.38	0.38	0.38	0.35
10	0.49	0.49	0.49	0.49	0.49	0.49	0.49	0.49	0.49	0.49	0.49	0.49	0.49	0.46
11	0.56	0.56	0.56	0.56	0.57	0.56	0.57	0.57	0.56	0.56	0.57	0.56	0.56	0.56
12	0.59	0.59	0.59	0.59	0.60	0.59	0.59	0.59	0.59	0.59	0.60	0.59	0.59	0.62
13	0.64	0.63	0.64	0.64	0.64	0.64	0.64	0.64	0.64	0.63	0.64	0.63	0.63	0.66
14	0.68	0.68	0.68	0.68	0.68	0.68	0.68	0.68	0.68	0.68	0.68	0.68	0.68	0.69
15	0.75	0.75	0.75	0.75	0.83	0.75	0.75	0.75	0.75	0.75	0.75	0.75	0.75	0.77
16	0.86	0.85	0.85	0.86	0.86	0.86	0.86	0.86	0.86	0.85	0.86	0.85	0.86	0.85
17	1.00	1.00	1.00	1.00	1.00	1.00	1.00	1.00	1.00	1.00	1.00	1.00	1.00	1.00
18	1.06	1.06	1.06	1.06	1.06	1.06	1.06	1.06	1.06	1.06	1.06	1.06	1.06	1.08
19	1.10	1.10	1.10	1.10	1.10	1.10	1.10	1.10	1.10	1.10	1.10	1.10	1.10	1.13
20	1.15	1.15	1.15	1.15	1.15	1.15	1.15	1.15	1.15	1.15	1.15	1.15	1.15	1.16
21	1.20	1.20	1.20	1.20	1.20	1.20	1.20	1.20	1.20	1.20	1.20	1.20	1.20	1.23
22	1.24	1.24	1.24	1.24	1.24	1.24	1.24	1.24	1.24	1.24	1.24	1.24	1.24	1.26
23	1.30	1.30	1.30	1.29	1.30	1.30	1.30	1.30	1.30	1.30	1.29	1.30	1.30	1.33
24	1.34	1.34	1.34	1.34	1.34	1.34	1.34	1.34	1.34	1.34	1.34	1.34	1.34	1.36

25	1.36	1.36	1.37	1.36	1.36	1.37	1.37	1.36	1.36	1.36	1.36	1.37	1.37	1.40
26	1.50	1.50	1.50	1.49	1.49	1.50	1.50	1.50	1.50	1.49	1.49	1.50	1.49	1.52
27	1.70	1.70	1.71	1.70	1.70	1.71	1.70	1.70	1.70	1.70	1.70	1.70	1.70	1.73
28	1.76	1.76	1.76	1.76	1.76	1.76	1.76	1.76	1.76	1.76	1.76	1.76	1.76	1.82
29	1.80	1.80	1.80	1.80	1.80	1.80	1.80	1.80	1.80	1.80	1.80	1.80	1.80	1.84
30	1.84	1.84	1.84	1.83	1.83	1.84	1.84	1.84	1.84	1.84	1.84	1.84	1.84	1.87
31	1.87	1.87	1.87	1.86	1.86	1.87	1.87	1.87	1.87	1.87	1.87	1.87	1.87	1.91
32	1.92	1.92	1.92	1.91	1.91	1.92	1.92	1.92	1.92	1.92	1.92	1.92	1.92	1.96
33	2.00	2.00	2.00	1.99	1.99	2.00	2.00	2.00	2.00	2.00	2.00	2.00	2.00	2.01
34	2.16	2.15	2.15	2.15	2.15	2.16	2.16	2.16	2.16	2.15	2.16	2.16	2.15	2.18
35	2.20	2.20	2.20	2.19	2.19	2.20	2.20	2.20	2.20	2.20	2.20	2.20	2.19	2.24

Note: RPA was relative peak areas

Table S10. RPA of common peaks of twenty-eight batches of samples

Peak	S1	S2	S3	S4	S5	S6	S7	S8	S9	S10	S11	S12	S13	S14
1	0.18	0.43	0.24	0.07	0.04	0.12	0.07	0.28	0.06	0.28	0.10	0.09	0.10	0.05
2	0.05	0.10	0.05	0.03	0.00	0.02	0.02	0.05	0.01	0.05	0.01	0.01	0.02	0.01
3	0.17	0.14	0.13	0.07	0.00	0.02	0.03	0.05	0.02	0.12	0.05	0.04	0.05	0.02
4	0.04	0.05	0.01	0.02	0.00	0.01	0.01	0.04	0.01	0.02	0.01	0.01	0.01	0.01
5	0.05	0.14	0.04	0.01	0.01	0.02	0.01	0.04	0.01	0.06	0.01	0.01	0.01	0.00
6	0.03	0.01	0.01	0.06	0.01	0.01	0.06	0.01	0.02	0.08	0.02	0.03	0.05	0.02
7	2.72	2.30	1.30	0.19	0.06	0.46	0.32	0.69	0.10	1.89	0.20	0.21	0.27	0.04
8	0.35	0.63	0.44	0.17	0.06	0.18	0.16	0.40	0.10	0.51	0.11	0.12	0.21	0.03
9	4.23	2.28	4.61	0.65	0.55	1.18	0.70	2.35	1.00	3.32	0.71	0.50	1.37	0.25
10	0.17	0.15	0.24	0.04	0.03	0.10	0.05	0.09	0.04	0.11	0.04	0.04	0.05	0.02
11	0.06	0.07	0.04	0.02	0.01	0.02	0.02	0.04	0.02	0.09	0.02	0.02	0.03	0.01
12	0.11	0.09	0.09	0.03	0.03	0.04	0.03	0.13	0.04	0.10	0.03	0.03	0.03	0.02

13	0.21	0.18	0.13	0.07	0.05	0.08	0.07	0.14	0.04	0.30	0.08	0.07	0.08	0.04
14	0.06	0.19	0.21	0.23	0.18	0.18	0.22	0.15	0.15	0.15	0.18	0.18	0.23	0.18
15	0.01	0.10	0.17	0.11	0.05	0.10	0.11	0.10	0.04	0.53	0.09	0.07	0.08	0.03
16	1.41	1.75	1.17	0.16	0.15	0.44	0.21	0.99	0.25	0.57	0.08	0.11	0.08	0.06
17	1.00	1.00	1.00	1.00	1.00	1.00	1.00	1.00	1.00	1.00	1.00	1.00	1.00	1.00
18	4.24	9.93	6.74	3.38	6.78	6.99	3.74	4.58	8.50	5.71	6.01	6.55	3.99	5.38
19	4.63	4.40	6.43	0.87	0.52	1.56	0.87	3.94	1.19	3.00	0.92	0.61	1.26	0.29
20	5.02	3.12	4.96	0.90	1.95	3.16	1.09	5.36	2.55	4.74	2.26	1.78	1.95	0.79
21	0.11	0.19	0.36	0.04	0.02	0.09	0.05	0.06	0.02	0.15	0.05	0.06	0.03	0.01
22	0.14	0.24	0.24	0.16	0.09	0.16	0.15	0.15	0.09	0.13	0.12	0.11	0.15	0.08
23	0.27	0.19	0.27	0.42	0.16	0.22	0.40	0.15	0.11	0.13	0.21	0.21	0.22	0.23
24	1.39	0.69	0.92	1.15	0.58	0.88	1.15	0.76	0.87	0.74	1.00	1.00	0.85	0.93
25	0.01	0.03	0.03	0.01	0.00	0.01	0.01	0.01	0.00	0.03	0.01	0.01	0.01	0.00
26	0.64	1.87	0.45	0.07	0.19	0.47	0.10	0.50	0.17	0.41	0.20	0.18	0.23	0.04
27	0.27	0.21	0.21	0.25	0.06	0.13	0.02	0.08	0.09	0.21	0.14	0.14	0.11	0.10
28	7.10	5.92	6.32	1.16	1.75	4.05	0.24	7.42	2.84	6.82	2.81	2.40	2.32	0.92
29	0.28	0.15	0.05	0.06	0.11	0.11	1.47	0.25	0.12	0.23	0.11	0.07	0.09	0.04
30	0.18	0.19	0.14	0.19	0.32	0.23	0.05	0.17	0.12	0.17	0.10	0.10	0.11	0.12
31	0.04	0.03	0.06	0.02	0.01	0.04	0.16	0.03	0.05	0.04	0.01	0.01	0.01	0.01
32	0.37	0.78	0.38	0.04	0.02	0.16	0.08	0.12	0.06	0.69	0.08	0.10	0.12	0.01
33	0.17	0.08	0.28	0.02	0.01	0.15	0.03	0.09	0.07	0.10	0.02	0.02	0.02	0.01
34	0.18	0.13	0.24	0.04	0.04	0.05	0.06	0.13	0.04	0.23	0.04	0.02	0.03	0.02
35	0.35	0.10	0.11	0.05	0.17	0.10	0.06	0.15	0.07	0.43	0.09	0.05	0.15	0.03

RPA of common peaks of twenty-eight batches of samples (continuation table)

**Peak**	**S15**	**S16**	**S17**	**S18**	**S19**	**S20**	**S21**	**S22**	**S23**	**S24**	**S25**	**S26**	**S27**	**S28**

1	0.05	0.05	0.08	0.08	0.05	0.03	0.05	0.12	0.09	0.10	0.07	0.11	0.06	0.30
2	0.01	0.01	0.03	0.01	0.01	0.01	0.01	0.02	0.02	0.02	0.01	0.02	0.01	0.05
3	0.02	0.02	0.02	0.03	0.02	0.04	0.03	0.05	0.05	0.03	0.04	0.04	0.02	0.03
4	0.01	0.01	0.00	0.00	0.01	0.01	0.01	0.01	0.01	0.00	0.01	0.01	0.00	0.01
5	0.00	0.00	0.02	0.01	0.00	0.00	0.01	0.02	0.01	0.01	0.01	0.01	0.02	0.11
6	0.02	0.08	0.03	0.04	0.08	0.08	0.07	0.12	0.10	0.11	0.12	0.04	0.01	3.48
7	0.04	0.17	0.69	0.33	0.11	0.03	0.14	0.31	0.12	0.77	0.25	0.56	0.12	0.26
8	0.03	0.31	0.34	0.20	0.14	0.13	0.19	0.24	0.18	0.31	0.26	0.23	0.06	0.07
9	0.25	0.56	0.33	1.09	0.22	0.20	0.60	1.41	0.44	0.63	0.61	0.80	0.56	0.70
10	0.02	0.02	0.02	0.03	0.02	0.01	0.02	0.07	0.03	0.04	0.03	0.06	0.02	0.09
11	0.01	0.02	0.01	0.02	0.01	0.01	0.02	0.04	0.02	0.03	0.03	0.03	0.02	0.19
12	0.02	0.02	0.03	0.03	0.02	0.01	0.02	0.02	0.02	0.03	0.02	0.04	0.01	0.02
13	0.04	0.04	0.09	0.05	0.05	0.05	0.04	0.06	0.07	0.06	0.08	0.07	0.05	0.03
14	0.18	0.18	0.14	0.15	0.20	0.36	0.21	0.29	0.21	0.16	0.25	0.18	0.12	0.09
15	0.03	0.03	0.01	0.04	0.05	0.03	0.04	0.04	0.11	0.03	0.18	0.04	0.01	0.07
16	0.06	0.13	0.21	0.13	0.12	0.07	0.17	0.25	0.10	0.22	0.15	0.25	0.08	0.39
17	1.00	1.00	1.00	1.00	1.00	1.00	1.00	1.00	1.00	1.00	1.00	1.00	1.00	1.00
18	5.38	5.92	12.71	7.14	2.59	1.94	4.10	3.95	3.24	6.30	2.63	4.79	9.38	2.46
19	0.29	0.63	0.51	1.18	0.39	0.32	0.81	1.45	0.58	0.81	0.57	0.76	0.54	1.39
20	0.79	0.93	0.57	1.35	0.65	0.49	1.01	3.60	0.95	1.47	1.53	1.48	1.00	0.02
21	0.01	0.01	0.04	0.01	0.02	0.01	0.01	0.04	0.04	0.05	0.02	0.01	0.03	0.08
22	0.08	0.08	0.06	0.07	0.13	0.13	0.13	0.18	0.12	0.14	0.13	0.19	0.06	0.04
23	0.23	0.18	0.07	0.09	0.33	0.03	0.19	0.23	0.28	0.17	0.33	0.17	0.08	0.18
24	0.93	0.91	0.54	0.54	0.63	0.01	0.67	0.77	0.64	0.64	0.67	0.69	0.49	0.35
25	0.00	0.00	0.01	0.00	0.01	0.00	0.00	0.01	0.01	0.02	0.01	0.01	0.01	0.01
26	0.04	0.05	0.14	0.06	0.09	0.04	0.13	0.15	0.05	0.15	0.19	0.09	0.03	0.34

27	0.10	0.09	0.10	0.07	0.15	0.01	0.13	0.18	0.16	0.18	0.18	0.08	0.06	2.48
28	0.92	1.08	1.07	1.85	1.11	0.76	1.55	3.59	1.28	1.91	2.33	2.28	1.09	0.02
29	0.04	0.07	0.03	0.05	0.08	0.04	0.10	0.16	0.05	0.07	0.14	0.11	0.05	0.23
30	0.12	0.06	0.06	0.07	0.18	0.11	0.11	0.05	0.14	0.09	0.17	0.53	0.06	0.00
31	0.01	0.01	0.02	0.01	0.03	0.00	0.01	0.02	0.02	0.02	0.03	0.05	0.00	0.03
32	0.01	0.08	0.38	0.10	0.01	0.01	0.05	0.11	0.04	0.28	0.03	0.08	0.09	0.05
33	0.01	0.01	0.02	0.01	0.02	0.00	0.01	0.04	0.02	0.03	0.03	0.05	0.01	0.20
34	0.02	0.02	0.28	0.02	0.03	0.02	0.04	0.06	0.05	0.06	0.06	0.02	0.02	0.07
35	0.03	0.06	0.03	0.04	0.08	0.07	0.10	0.18	0.06	0.27	0.12	0.11	0.02	0.00

Table S11. Eigenvalues and contribution rates of twenty-eight samples.

**Principal components**	**Initial eigenvalue**	**Variance contribution rate (%)**	**Cumulative contribution rate (%)**
1	7.83	22.37	22.37
2	5.78	16.52	38.90
3	5.04	14.41	53.30
4	3.11	8.87	62.17
5	2.54	7.27	69.44
6	2.03	5.80	75.24
7	1.61	4.61	79.85
8	1.35	3.85	83.70
9	1.14	3.27	86.96

Table S12. Loading matrix analysis results of 35 common peaks' principal components in twenty-eight samples.

Common peak	Loading matrix								
	PC1	PC2	PC3	PC4	PC5	PC6	PC7	PC8	PC9
1	-0.04	-0.09	0.72	0.11	0.43	0.12	-0.31	0.14	-0.14
2	0.08	0.18	0.84	-0.20	-0.22	-0.08	-0.02	0.00	0.21
3	0.61	0.10	0.32	0.01	0.03	-0.65	0.04	0.09	0.05
4	0.60	-0.10	0.18	0.19	-0.09	-0.46	-0.26	-0.01	0.24
5	-0.71	-0.19	0.53	0.00	0.23	0.06	0.10	-0.02	-0.02
6	-0.40	-0.50	0.56	0.29	0.34	0.04	0.19	-0.08	0.01
7	-0.22	0.66	0.34	-0.44	0.07	0.02	0.02	-0.05	-0.06
8	0.44	0.31	0.36	-0.39	0.26	-0.04	-0.06	-0.30	-0.13
9	-0.09	0.74	-0.11	0.31	0.12	-0.30	-0.18	-0.26	-0.19
10	-0.06	0.73	0.23	0.54	0.13	0.03	0.07	-0.01	0.02
11	-0.25	-0.38	0.55	0.29	0.52	0.06	-0.08	-0.16	-0.21
12	0.46	0.55	-0.10	0.04	0.18	0.36	-0.08	-0.24	0.20
13	0.77	0.20	0.22	-0.36	0.06	-0.07	0.08	0.09	0.04
14	0.85	-0.22	0.05	-0.01	0.17	-0.15	0.32	-0.10	-0.06
15	0.47	0.08	0.22	0.31	-0.19	-0.10	-0.08	0.50	-0.03
16	-0.27	0.70	0.10	0.09	-0.03	-0.08	0.36	-0.31	0.23
17	0.74	-0.34	0.13	0.03	0.11	-0.27	0.44	-0.04	-0.05
18	0.84	-0.22	0.06	-0.05	0.21	0.19	0.05	-0.22	0.04
19	-0.18	0.74	0.02	0.34	0.03	-0.41	-0.06	-0.28	-0.04
20	0.25	0.53	-0.50	0.31	0.32	0.21	-0.13	0.10	-0.15
21	-0.15	0.61	0.41	0.22	-0.11	-0.15	-0.02	0.34	0.10
22	0.91	-0.03	0.11	0.13	0.12	-0.05	0.23	-0.02	-0.04
23	0.68	-0.08	0.26	0.36	-0.20	0.27	-0.28	0.05	0.21

24	0.60	0.04	0.12	0.26	-0.04	0.41	-0.51	-0.19	0.20
25	0.42	0.25	0.59	-0.18	-0.02	0.03	-0.13	0.38	-0.17
26	-0.31	0.37	-0.20	0.06	0.12	0.27	0.37	0.41	0.22
27	-0.33	-0.48	0.58	0.31	0.39	0.08	-0.01	0.01	0.19
28	0.20	0.50	-0.60	0.05	0.46	-0.05	-0.16	0.20	0.02
29	0.22	0.17	0.35	0.33	-0.58	0.34	0.20	-0.10	-0.37
30	0.56	-0.04	-0.26	0.14	0.26	0.32	0.27	0.00	0.33
31	0.22	0.24	0.38	0.33	-0.60	0.35	0.24	-0.09	-0.24
32	-0.16	0.54	0.39	-0.67	0.12	0.16	-0.11	0.01	-0.08
33	-0.54	0.35	0.29	0.45	0.08	-0.06	0.25	0.07	0.26
34	0.01	0.36	0.42	-0.61	-0.07	0.20	0.11	-0.03	0.29
35	0.38	0.36	-0.10	-0.06	0.52	0.20	0.25	0.20	-0.34

Note: PC was principal components

Table S13. Score coefficient matrix of the chemical constituents.

**Common peaks**	**Principal components**								
	**PC1**	**PC2**	**PC3**	**PC4**	**PC5**	**PC6**	**PC7**	**PC8**	**PC9**
1	-0.07	0.20	-0.04	0.05	0.07	0.15	-0.04	0.15	-0.12
2	0.02	0.01	0.03	0.14	0.06	-0.21	-0.02	0.07	0.06
3	0.17	-0.02	0.12	-0.01	-0.13	-0.12	-0.13	0.16	-0.05
4	0.06	-0.04	0.11	-0.06	0.11	-0.21	-0.18	0.14	-0.06
5	-0.01	0.16	0.00	0.05	-0.06	0.01	0.01	-0.04	0.04
6	0.07	0.22	0.03	-0.04	-0.03	0.00	0.02	-0.08	0.06
7	-0.02	-0.01	0.03	0.19	-0.05	0.04	0.01	-0.03	-0.03
8	0.09	0.04	0.05	0.16	0.01	0.05	-0.01	-0.15	-0.19
9	0.00	0.00	0.24	-0.04	-0.02	0.07	0.00	-0.06	-0.24
10	0.01	0.07	0.18	-0.04	0.04	0.06	0.06	0.03	0.08

11	0.03	0.26	0.04	-0.02	0.00	0.13	0.02	-0.08	-0.16
12	0.00	-0.01	0.07	0.08	0.26	0.01	-0.03	-0.17	0.07
13	0.11	-0.04	-0.04	0.12	-0.02	0.00	-0.04	0.06	0.04
14	0.23	0.02	0.02	-0.02	-0.10	0.02	0.04	-0.08	0.02
15	-0.03	-0.03	-0.06	-0.07	-0.04	0.05	0.03	0.38	0.08
16	0.11	-0.02	0.21	0.05	0.01	-0.15	0.02	-0.22	0.17
17	0.26	0.03	0.02	-0.04	-0.18	-0.02	0.04	-0.05	0.06
18	0.12	0.05	-0.02	0.03	0.12	0.02	0.01	-0.16	-0.01
19	0.04	-0.01	0.28	-0.04	-0.02	-0.06	-0.03	-0.07	-0.15
20	-0.06	0.00	0.03	-0.06	0.06	0.27	0.01	0.04	-0.02
21	-0.05	-0.01	0.07	0.01	-0.03	-0.03	-0.02	0.27	0.13
22	0.18	0.02	0.02	-0.02	-0.04	0.05	0.05	-0.01	0.04
23	-0.06	0.01	-0.03	-0.04	0.29	-0.08	0.02	0.11	0.05
24	-0.12	0.02	0.00	0.01	0.42	-0.05	-0.03	-0.03	-0.08
25	-0.03	0.02	-0.10	0.10	-0.07	0.13	0.05	0.29	-0.04
26	-0.01	-0.02	-0.06	0.01	-0.04	0.07	-0.03	0.11	0.43
27	0.02	0.22	0.01	-0.03	0.11	-0.05	-0.10	0.00	0.11
28	-0.03	-0.03	0.03	-0.02	0.04	0.19	-0.19	0.10	0.03
29	-0.01	-0.02	-0.01	-0.03	-0.07	0.08	0.45	-0.04	-0.10
30	0.11	0.02	-0.03	-0.01	0.16	-0.01	-0.07	-0.11	0.32
31	-0.01	-0.03	0.00	-0.02	-0.03	0.02	0.41	-0.04	-0.01
32	-0.06	0.00	-0.06	0.25	-0.01	0.08	-0.02	-0.01	-0.07
33	0.03	0.08	0.14	-0.05	0.01	-0.10	-0.03	0.03	0.27
34	0.01	-0.04	-0.05	0.24	0.09	-0.14	-0.05	-0.07	0.19
35	0.08	0.08	-0.06	0.04	-0.17	0.37	0.09	0.03	0.02

Table S14. Principal component score result

Samples	PCS									Y	Rank
	y1	y2	y3	y4	y5	y6	y7	y8	y9		
S1	-0.20	-0.62	2.08	0.43	-0.58	-0.68	-0.58	-1.03	0.31	0.06	10
S2	-1.17	-0.91	-1.04	-0.23	-1.48	-1.23	-0.01	-0.42	0.49	-0.88	28
S3	-0.56	-0.27	3.21	0.05	-0.66	-0.48	-0.27	0.67	0.55	0.29	7
S4	1.22	0.23	1.06	0.09	1.77	-2.01	-1.03	2.07	0.11	0.60	3
S5	0.35	-0.38	-0.22	-0.49	1.05	2.27	0.14	-1.16	2.51	0.22	8
S6	-0.70	-0.26	0.73	-0.45	-0.11	0.32	-0.18	0.31	2.27	-0.05	14
S7	0.14	-0.29	0.54	0.23	0.46	-0.45	4.86	0.71	-0.33	0.38	6
S8	-1.08	-1.09	-0.39	-0.70	-0.58	-0.81	-0.18	-0.99	-0.44	-0.79	27
S9	-0.55	-0.58	0.66	-0.89	0.47	0.19	0.08	-1.15	0.27	-0.22	23
S10	-0.81	-0.22	-0.04	0.71	-1.06	0.71	-0.33	1.31	-0.47	-0.20	22
S11	-0.59	-0.16	-0.46	-0.36	0.29	0.83	-0.61	1.39	-0.13	-0.19	21
S12	-0.53	-0.07	-0.55	-0.20	0.47	0.55	-0.63	1.31	0.10	-0.16	20
S13	-0.15	0.01	0.09	-0.37	-0.40	0.74	-0.31	0.52	-0.89	-0.07	15
S14	0.16	-0.11	-0.92	-0.75	1.71	-0.30	-0.26	-0.29	-0.18	-0.12	18
S15	0.16	-0.11	-0.92	-0.75	1.71	-0.30	-0.26	-0.29	-0.18	-0.12	19
S16	0.92	0.79	1.07	0.83	1.96	0.57	-0.43	-1.38	-2.01	0.69	2
S17	-0.32	-0.36	-1.06	4.03	0.26	-1.34	-0.33	-0.82	0.94	0.00	13
S18	-0.53	-0.48	0.14	-0.32	-0.40	-0.09	-0.17	-0.97	-1.77	-0.39	25
S19	0.71	-0.13	-1.19	-0.17	0.29	-0.33	0.44	0.32	0.83	0.02	12
S20	4.15	-0.28	-0.23	-0.31	-2.30	-0.11	-0.22	-0.44	0.09	0.72	1
S21	0.17	-0.57	-0.42	-0.57	-0.36	-0.40	0.03	-0.83	-0.33	-0.30	24
S22	-0.26	0.35	0.55	-0.41	-0.62	1.43	-0.06	0.21	-1.15	0.05	11
S23	0.20	-0.04	-0.72	-0.17	0.00	-0.75	-0.25	1.40	-0.53	-0.12	17

S24	0.03	1.07	-0.12	2.13	-0.59	2.41	0.12	0.65	-0.31	0.54	4
S25	0.32	-0.29	-0.78	-0.48	-0.39	0.23	-0.03	1.32	0.48	-0.10	16
S26	0.37	0.21	0.17	0.00	0.47	0.37	0.21	-1.32	0.40	0.20	9
S27	-0.76	-0.04	-1.01	-0.10	-0.55	-0.16	0.08	-0.45	-1.39	-0.51	26
S28	-0.67	4.58	-0.25	-0.79	-0.82	-1.16	0.16	-0.66	0.76	0.44	5

Note: PCS was principal component score, y was nine principal component score, Y was comprehensive score of PR samples.

Table S15. VIP values of 35 chromatographic peaks with antioxidant activity

Peaks	VIP value		Peaks	VIP value	
	DPPH	ABTS		DPPH	ABTS
P1	1.26	0.98	P19	0.91	1.20
P2	0.89	0.75	P20	0.92	0.99
P3	0.95	0.91	P21	1.16	1.22
P4	1.33	1.27	P22	1.23	1.11
P5	0.80	0.22	P23	0.90	0.92
P6	1.16	1.19	P24	1.04	1.05
P7	0.62	0.39	P25	1.07	0.94
P8	1.28	1.01	P26	1.32	1.54
P9	0.95	1.12	P27	1.18	1.10
P10	0.83	1.04	P28	0.92	0.82
P11	1.21	1.06	P29	0.69	0.76
P12	1.14	1.17	P30	0.66	1.01
P13	1.08	1.02	P31	0.58	0.60
P14	1.24	1.20	P32	0.43	0.51
P15	0.91	1.02	P33	0.42	0.74
P16	0.67	0.88	P34	0.58	0.59
P17	1.31	1.30	P35	0.81	0.84
P18	1.34	1.24			

Table S16. Regression coefficients of 35 common peaks with antioxidant activity.

Peaks	Regression coefficient		Peaks	Regression coefficient	
	DPPH	ABTS		DPPH	ABTS
P1	-0.12	-0.05	P19	0.05	0.08
P2	-0.06	-0.04	P20	0.05	0.05
P3	0.06	0.01	P21	0.21	0.12
P4	0.19	0.06	P22	-0.12	-0.07
P5	-0.05	-0.02	P23	0.04	-0.02
P6	-0.08	-0.12	P24	0.09	0.01
P7	-0.09	0.00	P25	-0.14	-0.08
P8	-0.14	-0.06	P26	0.23	0.15
P9	0.02	0.07	P27	-0.05	-0.11
P10	0.04	0.06	P28	-0.05	0.03
P11	-0.11	-0.08	P29	0.12	0.07
P12	0.14	0.04	P30	-0.07	-0.09
P13	-0.01	-0.04	P31	0.05	0.05
P14	-0.09	-0.09	P32	-0.07	-0.03
P15	0.15	0.07	P33	0.03	0.07
P16	0.02	0.06	P34	-0.07	-0.06
P17	-0.13	-0.12	P35	-0.11	-0.06
P18	-0.11	-0.09			

Table S17. Twenty-eight batches of persicae ramulus from different areas.

Samples	Origin	Samples	Origin
S1	Dangwu Town, Guiyang City, Guizhou Province 1	S15	Guangshun Town, Changshun County, Guizhou Province
S2	Dangwu Town, Guiyang City, Guizhou Province 2	S16	Shenzhou, Hengshui City, Hebei Province
S3	Dangwu Town, Guiyang City, Guizhou Province 3	S17	Poxi Town, Jianhe County, Guizhou Province 2
S4	Dangwu Town, Guiyang City, Guizhou Province 4	S18	Nanshao Town, Jianhe County, Guizhou Province
S5	Dangwu Town, Guiyang City, Guizhou Province 5	S19	Gedong Town, Jianhe County, Guizhou Province
S6	Dangwu Town, Guiyang City, Guizhou Province 6	S20	Taiyong Town, Jianhe County, Guizhou Province
S7	Dangwu Town, Guiyang City, Guizhou Province 7	S21	Mindong Township, Jianhe County, Guizhou Province
S8	Dangwu Town, Guiyang City, Guizhou Province 8	S22	Nanjia Town, Jianhe County, Guizhou Province
S9	Wangmo County, Xingyi City, Guizhou Province 1	S23	Liuchuan Town, Jianhe County, Guizhou Province
S10	Poxi Town, Jianhe County, Guizhou Province 1	S24	Nanzhai Town, Jianhe County, Guizhou Province
S11	Wangmo County, Xingyi City, Guizhou Province	S25	Jiuyang Town, Jianhe County, Guizhou Province
S12	Jinsha County, Bijie City, Guizhou Province 2	S26	Nanming Town, Jianhe County, Guizhou Province
S13	Dayegou, Bijie City, Guizhou Province	S27	Guanmo Town, Jianhe County, Guizhou Province
S14	Wangmo County, Xingyi City, Guizhou Province 3	S28	Anguo Pharmaceutical City, Baoding City, Hebei Province

Table S18. Factors and levels of process optimization of RSM.

factor	levels		
	-1	0	1
extraction time A（min）	40	50	60
liquid-to-solid ratio B（g/mL）	1:20	1:25	1:30
concentration of ethanol C（%）	40	50	60

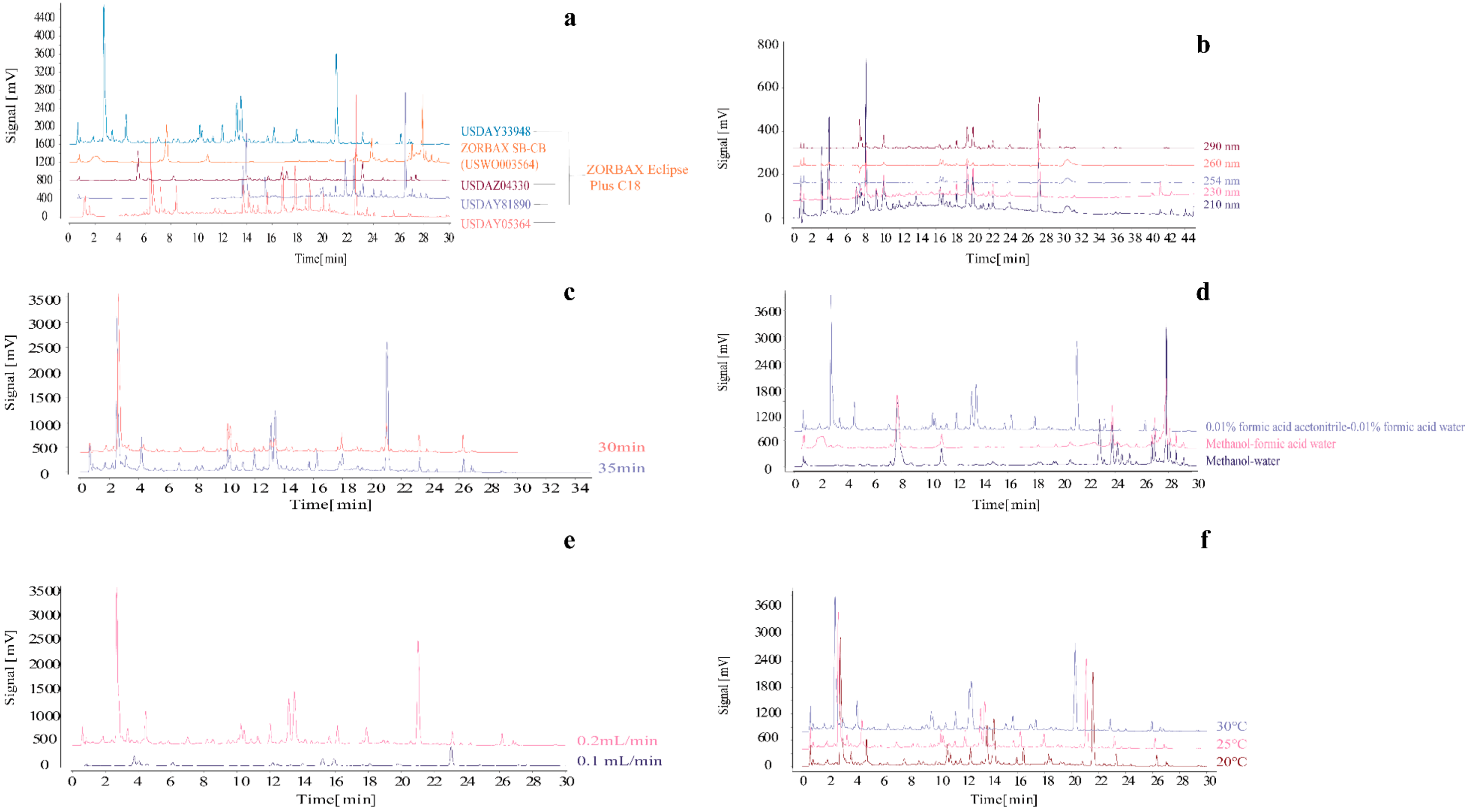

Figure S1. System suitability investigation results (UPLC conditions of persicae ramulus; **a**: column; **b**: wavelength; **c**: analysis time; **d**: mobile phase; **e**: flow rate; **f**: column temperature)

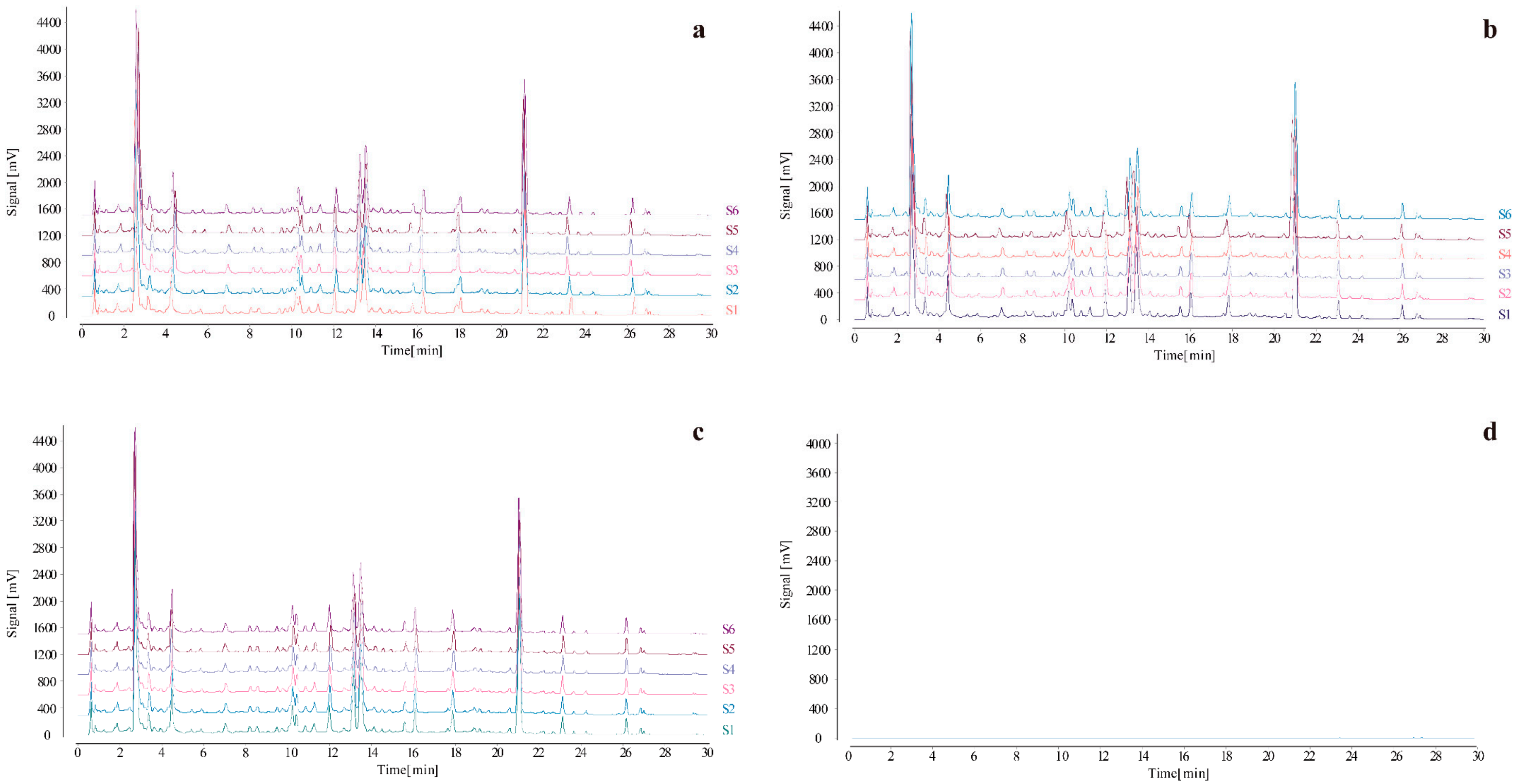

Figure S2. Methodology validation of UPLC (**a**: precision; **b**: reproducibility; **c**: stability; **d**: blank)

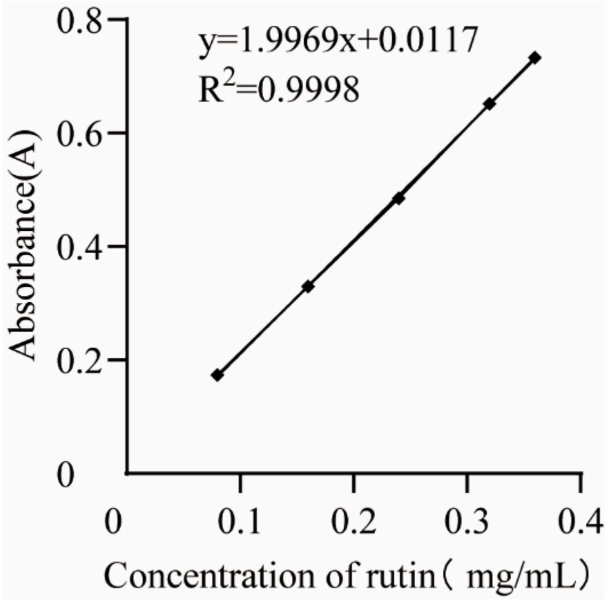

Figure S3. Standard curve
